# Mutual repression between Gbx2 and Otx2 in sensory placodes reveals a general mechanism for ectodermal patterning

**DOI:** 10.1016/j.ydbio.2012.04.025

**Published:** 2012-07-01

**Authors:** Ben Steventon, Roberto Mayor, Andrea Streit

**Affiliations:** a Department of Craniofacial Development, King's College London, Guy's Campus, Tower Wing Floor 27, London SE1 9RT, UK; b Department of Cell and Developmental Biology, University College London, Gower Street, London WC1E 6BT, UK

**Keywords:** Chick, Cranial ganglia, Ear, Eye, Fate map, Placodes, Trigeminal, *Xenopus*

## Abstract

In the vertebrate head, central and peripheral components of the sensory nervous system have different embryonic origins, the neural plate and sensory placodes. This raises the question of how they develop in register to form functional sense organs and sensory circuits. Here we show that mutual repression between the homeobox transcription factors Gbx2 and Otx2 patterns the placode territory by influencing regional identity and by segregating inner ear and trigeminal progenitors. Activation of Otx2 targets is necessary for anterior olfactory, lens and trigeminal character, while Gbx2 function is required for the formation of the posterior otic placode. Thus, like in the neural plate antagonistic interaction between Otx2 and Gbx2 establishes positional information thus providing a general mechanism for rostro-caudal patterning of the ectoderm. Our findings support the idea that the Otx/Gbx boundary has an ancient evolutionary origin to which different modules were recruited to specify cells of different fates.

## Highlights

► Otx2 and Gbx2 segregate placode progenitors of different fates. ► Otx2 and Gbx2 mutually repress each other. ► Otx2 target activation is required for olfactory, lens and trigeminal specification. ► Gbx2 is required for otic placode specification.

## Introduction

In the vertebrate head, placodes give rise to crucial parts of the sensory nervous system including the olfactory epithelium, the lens, the inner ear and the sensory neurons of the cranial ganglia (Baker and Bronner-Fraser, 2001; Streit, 2007; Schlosser, 2010). They form at discrete positions outside of the central nervous system, with which they build complete sense organs and sensory circuits. How are central and peripheral components aligned? Here we explore the possibility that a common molecular mechanism allocates anterior-posterior positional information across the entire ectoderm.

At neurula stages, placode precursors occupy a unique territory, the pre-placodal region (PPR), where cells of different placodal fates are interspersed (Kozlowski et al., 1997; Streit, 2002; Bhattacharyya et al., 2004; Xu et al., 2008; Pieper et al., 2011); their anterior-posterior identity is not fully specified (Henry and Grainger, 1987; Gallagher et al., 1996; Grainger et al., 1997; Baker et al., 1999; Groves and Bronner-Fraser, 2000; Baker and Bronner-Fraser, 2000; Bhattacharyya et al., 2004; Bailey et al., 2006; Bhattacharyya and Bronner-Fraser, 2008). Although some placode inducing signals have been identified (McCabe and Bronner-Fraser, 2009; Ladher et al., 2010; Schlosser, 2010), additional cell intrinsic mechanisms must exist that determine the interpretation of such signals and mediate PPR subdivision. In the neural tube, mutual repression between pairs of transcription factors establishes boundaries to segregate cells of different fates (Broccoli, et al., 1999; Millet, et al., 1999; Katahira, et al., 2000; Li and Joyner, 2001; Kobayashi et al., 2000; Nakamura and Watanabe, 2005). One of the best-studied interactions is that between Otx2 and Gbx2. While *Gbx*2 is first detected within the posterior neuroectoderm, *Otx*2 becomes restricted anteriorly (Simeone et al., 1992, 1993; von Bubnoff et al., 1996; Tour et al., 2001). Both factors mutually repress each other to form a sharp border (Millet et al., 1999; Katahira et al., 2000; Tour et al., 2002a; Glavic et al., 2002) and this interaction establishes the midbrain-hindbrain boundary (MHB) (Wassarman et al., 1997; Acampora et al., 1995, 1997, 1998; Rhinn et al., 1998; Broccoli, et al., 1999; Li et al., 2005). However, in the absence of Otx2 or Gbx2 function MHB specific genes remain expressed, but are mislocalized. These observations suggest that the Otx2/Gbx2 interface is primarily important for positioning the MHB (Li and Joyner, 2001; Raible and Brand, 2004).

Does a similar mechanism establish regional identity within the PPR? Some neural plate border derivatives depend on Gbx2 and Otx2 function. Gbx2 is required for neural crest cell formation and transcripts are also found in the PPR (Li et al., 2009). In mice, Gbx2 is necessary for otic vesicle morphogenesis after placode formation (Lin et al., 2005). Anteriorly, Otx2 cooperates with Notch signaling to promote lens fate (Ogino et al., 2008), while loss of Otx2 function in mice results in lens, olfactory and inner ear defects (Acampora et al., 1995). However, due to the severe head phenotypes in these mutants it is difficult to assess the specific requirement of Otx2 in placode formation.

Here, we test the hypothesis that Otx2 and Gbx2 provide a cell intrinsic mechanism to establish anterior-posterior positional information in sensory placode progenitors. We show that they mutually repress each other to form a boundary between prospective otic and trigeminal placodes and mediate cell segregation within the PPR. While Gbx2 is required for otic specification, Otx2 is necessary for the specification of the olfactory, lens and trigeminal placodes. Thus, Otx2 and Gbx2 provide a global mechanism for patterning of the embryonic ectoderm and ensure the coordinated development of the central and peripheral nervous system in the head.

## Materials and methods

### Embryo techniques

Fertile hens' eggs (Henry Stewart, UK) were incubated at 38 °C for 24–30 h until they had reached the appropriate stage (Hamburger and Hamilton, 1951; HH). Small groups of cells were labeled with DiI (Streit, 2002); the position of labeled cells along the anterior-posterior axis was expressed as a percentage of the distance from the center of Hensen's node to the anterior tip of the prechordal plate (hn-pc distance). The medio-lateral position was determined as a percentage of the distance between the midline and the edge of the neural plate (Fig. 3(A)). Embryos were grown in modified New culture (New, 1955; Stern and Ireland., 1981) until they reached HH12. The fate of labeled cells was determined by morphology or by colocalization with Pax3 for the ophthalmic trigeminal placode. To compare the cell fate to the position of the *Gbx*2*/Otx*2 boundary, stage HH7 embryos were processed for double in situ hybridization (ISH) for *Otx*2 and *Gbx*2. The gene expression boundary was determined and expressed as a percentage of the hn-pc distance (Fig. 3(A)). Using these measurements, DiI labels were classified as either within the *Gbx*2 or *Otx*2 domain.

*Xenopus* embryos were obtained as described previously (Gomez-Skarmeta et al., 1998) and staged according to Nieuwkoop and Faber (1967). D1 blastomeres of 8-cell or A3 blastomeres of 32-cell embryos were injected with RNA and fluorescein or rhodamine dextran (FDX; RDX) or with FDX alone (Aybar et al., 2003). Plasmids were linearized; RNA transcribed using SP6 or T7 RNA polymerases, and the GTP cap analog (Harland and Weintraub, 1985). Purified RNA was resuspended in DEPC-water and mixed with FDX to label the injected side. Full length nuclear *GFP*, nuclear *RFP*, *Otx*2 and *Gbx*2 mRNA, or *Gbx*2 morpholinos were used (Li et al., 2009). To repress or activate Gbx2 and Otx2 downstream targets, constructs in which their homeodomain was fused to the repressor domain of engrailed (*Otx*2*-EnR* and *Gbx*2*-EnR*) or the activator E1A were injected; fusion of these constructs to the glucocorticoid receptor (*Otx*2*-EnR-GR*, *Gbx*2*-EnR-GR*; Glavic et al., 2002) allows temporally controlled activation upon addition of dexamethasone (DEX; 10 μM). Embryos were then grown to the desired stage and processed for ISH and antibody staining. Embryos with the lineage tracer outside of the PPR or inside the neural plate were discarded unless otherwise stated.

### In situ hybridization and immunohistochemistry

For ISH, antisense digoxigenin (DIG) or fluorescein labeled RNA probes were used. *Xenopus* embryos were prepared, hybridized and stained as previously described (Harland, 1991), and NBT/BCIP or BCIP alone were used to reveal the signal. The genes analyzed were *Otx*2 (Blitz and Cho, 1995), *Gbx*2 (von Bubnoff et al., 1996), *Eya*1 (David et al., 2001), *Pax*8 (Heller and Brändli, 1999), *Pax*2 (Heller and Brändli, 1999), *Pax*3 (Bang et al., 1997), *Dmrt*4 (Huang et al., 2005), *Pax*6 (Hirsch and Harris, 1997), *Runx*3 (Park and Saint-Jeannet, 2010) and *FoxE*3 (Kenyon et al., 1999). FDX was visualized with an alkaline phosphatase conjugated anti-fluorescein antibody (AP-anti-FLU; Roche). Whole-mount ISH in chick (Streit et al., 1997) was performed with DIG labeled anti-sense probes for *Otx*2 (Bally-Cuif et al., 1995) and fluorescein-labeled probes for *Gbx*2 (Shamim and Mason, 1998). For double ISH, embryos were hybridized with both probes followed by consecutive antibody staining with alkaline phosphatase coupled-anti-DIG and anti-fluorescein antibodies (Roche) using fast red and NBT/BCIP for color development, respectively. Immunostaining on cryosections was performed (Bailey et al., 2006) using antibodies against Otx2 (Abcam; 1:50) and Pax3 (Developmental Hybridoma Bank; 1:10) and appropriate secondary antibodies (Invitrogen; 1:1000). Sections were imaged on a Leica TCS SP5 confocal microscope.

## Results

### *Otx*2 and *Gbx*2 form a boundary within the sensory placode territory

**Fig. 1:**
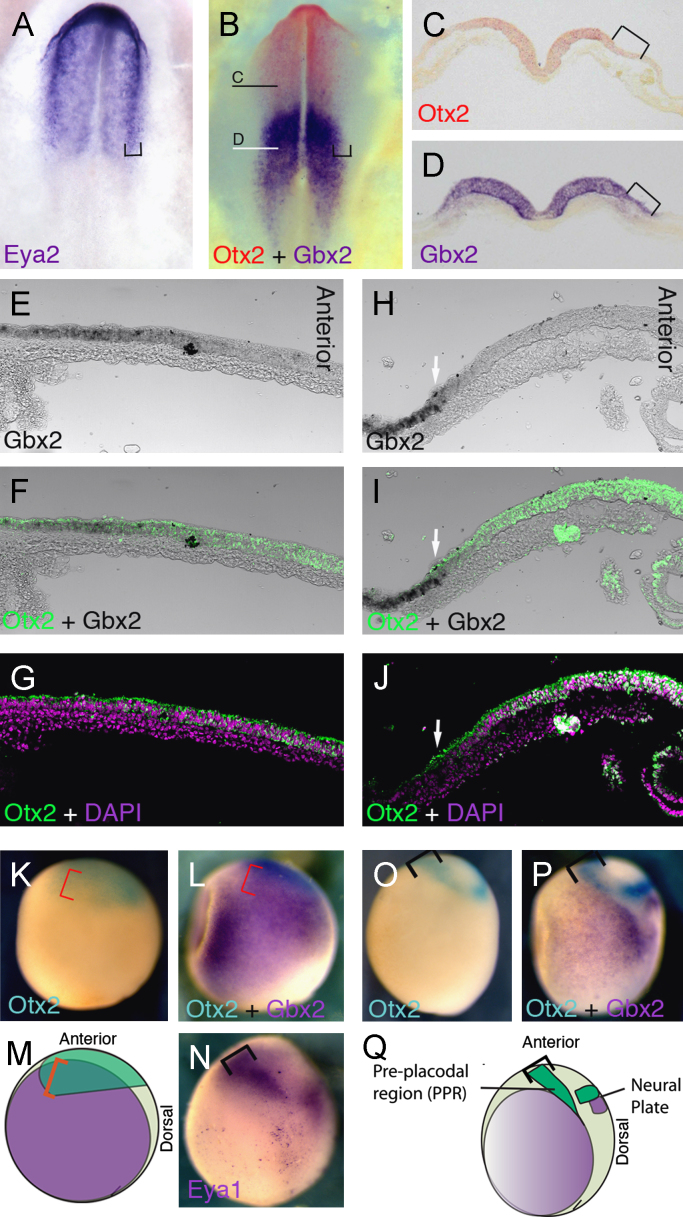
*Otx*2 and *Gbx*2 form a boundary within the PPR. (A) Expression of *Eya*2 marks the PPR (bracket). (B)–(D) Double ISH for *Otx*2 (red) and *Gbx*2 (blue) at HH7. Lines indicate the level of sections shown in (C) and (D). (E)–(J) Parasagittal sections of stage HH5 (E)–(G) and HH7 (H)–(J) chick embryos after *Gbx*2 ISH (black (E), (F), (H), (I)) and *Otx*2 immunostaining (green; (F), (G), (I), (J)); DAPI labels nuclei ((G), (J); magenta). Arrows in (H)–(J) indicate anterior limit of *Gbx*2. (K) and (L) Double ISH in *Xenopus* for *Otx*2 ((K), (L); turquoise) and *Gbx*2 ((L); blue) at stage 12, dorsal to right anterior to the top. Red brackets indicate overlapping expression. (M) Diagram summarising expression of *Otx*2 and *Gbx*2 at stage 12 in *Xenopus*; red bracket: PPR. (N) *Eya*1 at stage 13 labels the PPR (bracket). (O) and (P) Double ISH for *Otx*2 ((O), (P); turquoise) and *Gbx*2 ((P); blue) at stage 13, dorsal to the right. Black brackets indicate the PPR. (Q) Diagram summarising neural plate and PPR expression of *Otx*2 and *Gbx*2 at stage 13 in *Xenopus*.

In the neural tube *Otx*2 and *Gbx*2 expression initially overlaps but then resolves to form a boundary at the MHB (Simeone et al., 1993; von Bubnoff et al., 1996; Millet et al., 1999; Tour et al., 2002a; Glavic et al., 2002). In chick *Eya*2 expression identifies the PPR (Fig. 1(A); McLarren et al., 2003; Streit, 2007). While Otx2 and *Gbx*2 expression overlap in this territory at HH5 (Fig. 1(E)–(G)), both domains abut later (Fig. 1(B)–(D), (H)–(J)) with *Gbx*2 restricted to the posterior PPR. A similar boundary is observed in the *Xenopus* PPR (Fig. 1(K)–(P)). At stage 11.5, *Otx*2 encompasses both the anterior neural plate and its border (Fig. 1(K)), while *Gbx*2 is present posteriorly but overlapping with *Otx*2 (Fig. 1(L) and (M); red bracket). At neural plate stages, *Eya*1 demarcates the PPR (Fig. 1(N); black bracket); *Otx*2 and *Gbx*2 expression has resolved into a neural plate domain dorsally and a PPR domain laterally (Fig. 1(O) and (P); black bracket). In both regions, *Otx*2 and *Gbx*2 expression does not overlap (Fig. 1(Q)). Thus, like in the neural plate, *Gbx*2 and *Otx*2 form a gene expression boundary within the PPR in *Xenopus* and chick.

### Otx2 and Gbx2 segregate otic and trigeminal fates

**Fig. 2:**
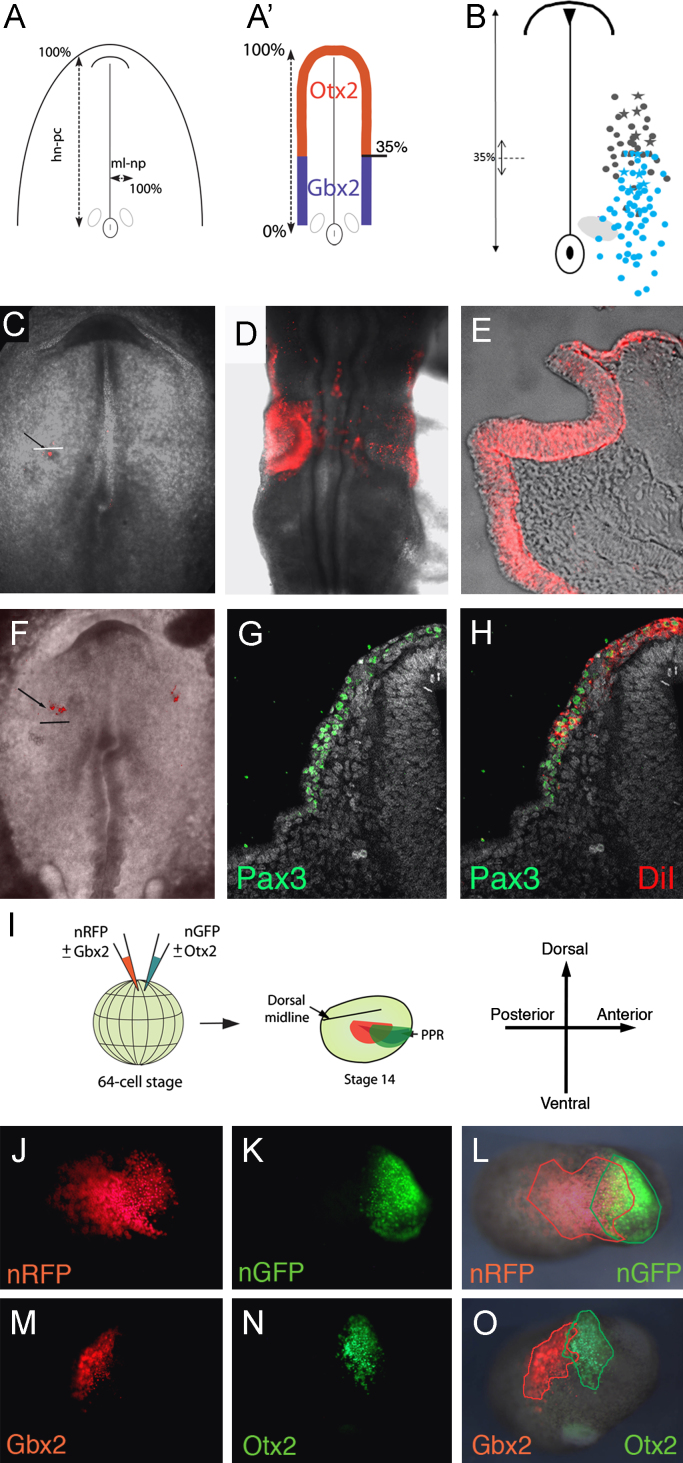
The *Otx*2*/Gbx*2 boundary separates otic and trigeminal precursors. (A) Diagram showing HH 7 stage chick embryo: the distance from the center of Hensen's node to the anterior tip of prechordal plate (hn-pc) and from the midline to the edge of the neural plate (ml-np) were set to 100%, respectively. The position of DiI label was measured and expressed as percentage of each distance. (A′). The position of the Gbx2/Otx2 boundary was measured using the same landmarks. In total 9 embryos were measured with the boundary on average at 35±7%; most anterior position measured: 42%, most posterior position: 24.5%. (B) Diagram combining labels from this study and published fate maps (Streit, 2002; Xu et al., 2008); gray: labels contributing to the trigeminal placode; blue: labels contributing to the otic placode. Circles: labels from published fate maps; squares: labels with dual fate from the current study; stars: labels from the current study. 35% indicates the average position of the Otx2/Gbx2 boundary (dotted line)±standard deviation (small arrow); note: mixed trigeminal and otic fates mostly locate near this boundary. (C) HH7 embryo with DiI labeled cells posterior to the average position of the *Otx*2/*Gbx*2 boundary (white line). (D) and (E) At HH12 their descendants contribute to the otic placode as shown in whole mount (D) and in transverse sections (E) and (F). HH7 embryo with DiI label anterior to the average position of the Otx2/Gbx2 boundary (black line). (G) and (H) At HH11 their descendants overlap with Pax3 protein (green) in the trigeminal placode. In total, 21 labels were placed into the Otx2^+^ and 12 into the Gbx2^+^ domain. (I) Diagram showing the experimental design: blastomeres were injected at the 64-cell stage in *Xenopus* and their position scored at stage 14. Arrows show the orientation of all embryos. (J)–(L) Neighboring blastomeres were injected with nGFP and nRFP and grown until stage 14. Descendants from injected cells are intermingled as indicated by red and green outlines in L (100%, *n*=10). (M)–(O) When injected with nGFP/*Otx*2 and nRFP/*Gbx*2 descendants from adjacent blastomeres do not mix (boundary in 79% of embryos, *n*=14). Red and green outlines in O show the distribution of cells.

Otx2 and Gbx2 have previously been implicated in the maintenance of compartment boundaries (Zervas et al., 2004; Sunmonu et al., 2011). Do they segregate progenitors of different fates in the PPR? At HH 7 in chick, the *Otx*2/*Gbx*2 boundary lies on average at 35% of the distance from Hensen's node to the anterior tip of the prechordal plate (Fig. 2(A) and (A'); 35±7%; most anterior: 42%, most posterior: 24.5%) roughly corresponding to the most anterior location of otic progenitors (Streit, 2002). DiI labeling shows that cells near the boundary contribute to two placodes: the otic and the trigeminal. The majority of labels that gave rise to both placodes are found close to this boundary, with the exception of two injections at around 20% i.e., within the *Gbx*2 territory. Anterior to the boundary cells mainly contribute to the maxillomandibular trigeminal placode (mmV) and to the ophthalmic Pax3^+^ (opV) trigeminal territory (Fig. 2(B), (F)–(H)). In contrast, posterior to the *Otx*2/*Gbx*2 border, cells mainly localize to the otic placode (Fig. 2(B)–(D)), but are occasionally found in the mmV (Fig. 2(B); Xu et al., 2008). However, except the two injections mentioned above their original location lies well within the most posterior position of the Otx2/Gbx2 boundary measured suggesting that these cells may indeed arise from the *Gbx*2 territory. It is therefore possible that due to differences in individual embryos, fate maps overestimate cell mixing (see Pieper et al., 2011 for discussion). In general, our current findings agree with previously published fate maps (Fig. 2(B); Streit, 2002; Xu et al., 2008). Thus, although no strict cell fate segregation is observed, the vast majority of trigeminal precursors come from the *Otx*2 region, while otic cells largely arise from *Gbx*2^+^ cells suggesting that in the PPR the *Otx*2/*Gbx*2 boundary roughly separates trigeminal and otic territories.

Can Otx2 and Gbx2 sort cells in the PPR? Using *Xenopus* we compared the behavior of control-injected cells with those carrying exogenous Gbx2 and Otx2. Descendents of the A3 blastomere, which gives rise to placodes, were injected at the 64-cell stage with mRNA encoding nuclear-*GFP* and nuclear-*RFP* alone or in combination with *Otx*2 and *Gbx*2, respectively (Fig. 2(I)). Significant overlap between GFP and RFP expressing cells is observed at stage 14 in controls (Fig. 2(J)–(L)). In contrast, cells in the PPR and future epidermis overexpressing *Gbx*2 form a boundary with cells expressing exogenous *Otx*2 with only some cells intermingling (Fig. 2(M)–(O)). Together these results show that Otx2 and Gbx2 control cell sorting and are part of the molecular mechanism that segregates sensory progenitors to different placodes.

### The *Otx*2/*Gbx*2 boundary in the PPR forms by a cross-repressive mechanism

**Fig. 3:**
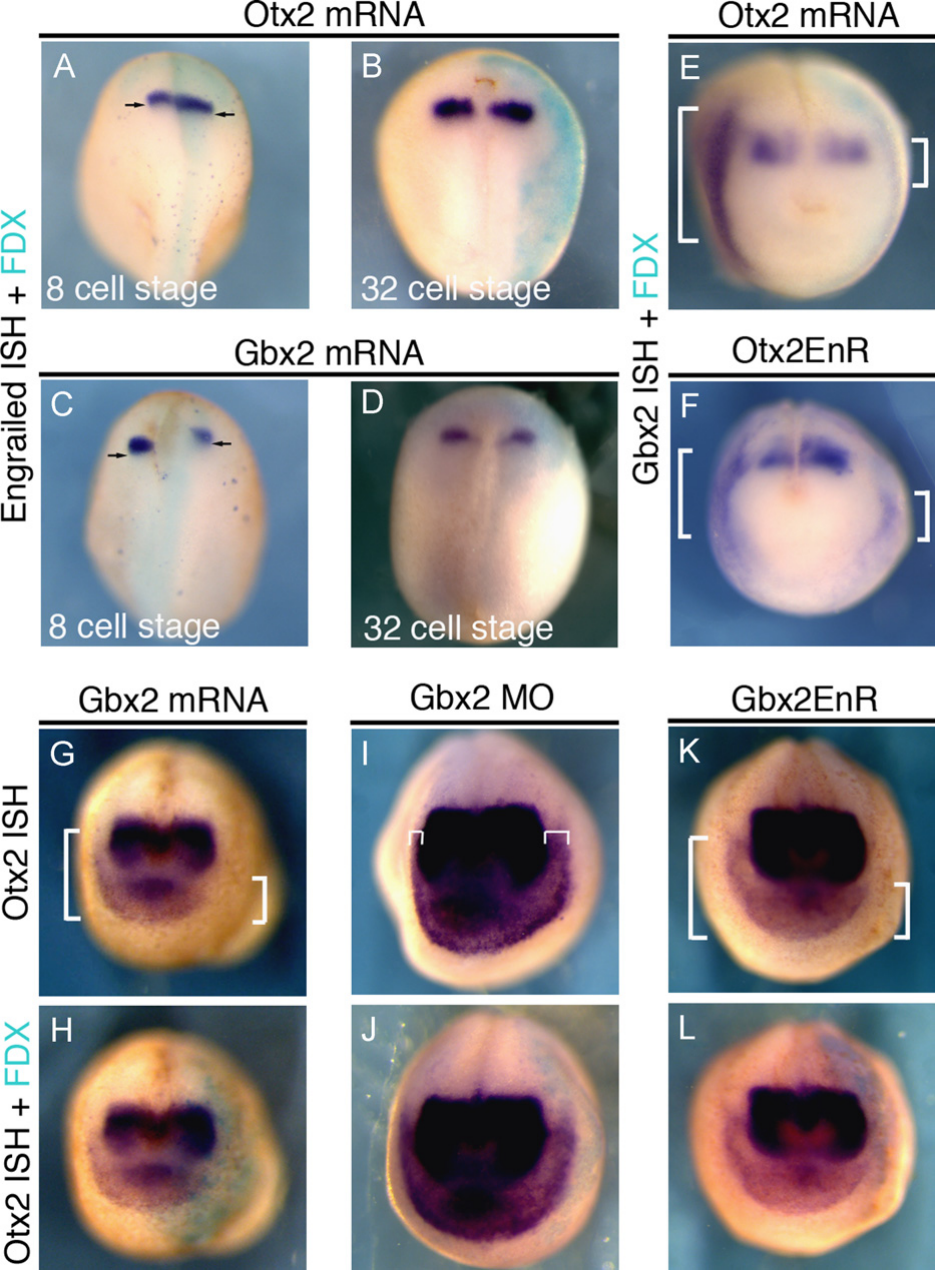
Otx2 and Gbx2 mutually repress each other in the PPR. (A) and (B) Injection of *Otx*2 mRNA into the D1 blastomere of 8-cell stage embryos (A) shifts *En-*1 posteriorly on the injected side (50%. *n*=28; FDX: turquoise), while injection into the A3 blastomere of a 32-cell stage embryo has little effect ((B); 5% affected, *n*=36). (C) and (D) Injection of *Gbx*2 mRNA into the D1 blastomere at 8-cell stage (C) shifts *En-*1 anteriorly on the injected side (68%; *n*=64; FDX: turquoise), while injection into the A3 blastomere at 32-cell stage has no effect ((D); 0% affected, *n*=31). Dorsal view, anterior to the top. (E) and (F) Injection of *Otx*2 (E; 68% affected, *n*=31) or *Otx*2*-EnR* ((F); 77% affected, *n*=17) into A3 at the 32-cell stage inhibits *Gbx*2 in the PPR. Compare bracket in the injected (FDX: turquoise) and uninjected side. (G) and (H) Injection of *Gbx*2 into A3 at the 32-cell stage leads to *Otx*2 repression (68%; *n*=26); compare brackets (G) on the injected ((H): FDX, turquoise) and uninjected side. (I) and (J) Co-injection of splice and translation blocking Gbx2 morpholinos into A3 at 32-cell stage leads to *Otx*2 expansion (73%; *n*=33); compare brackets (I) on the injected ((J) FDX, turquoise) and uninjected side. (K) and (L) Injection of *Gbx*2*-EnR* into A3 at the 32-cell stage leads to a loss of *Otx*2 in 62% of embryos (*n*=52); compare brackets (K) on the injected ((L) FDX, turquoise) and uninjected side. (E)–(L) Frontal view, dorsal to the top.

In the neural plate, Otx2 and Gbx2 mutually repress each other transcriptionally to form a sharp boundary (Millet et al., 1999; Katahira et al., 2000; Tour et al., 2002a; Glavic et al., 2002). To confirm this we injected *Otx*2 or *Gbx*2 mRNA into the D1 blastomere at the 8-cell stage targeting the neural plate and its border. As expected *Otx*2 misexpression shifts the MHB marker *En-*1 posteriorly (Fig. 3(A)), whereas misexpression of *Gbx*2 leads to an anterior shift (Fig. 3(C); see also: Tour et al., 2002b). In contrast, when *Otx*2 or *Gbx*2 mRNA is targeted to the PPR (A3 blastomere injection at 32-cell stage) changes in neural *En-*1 expression are rarely observed (Fig. 3(B) and (D)). This approach therefore allows us to analyze the role of these transcription factors specifically in placode progenitors, without interfering with neural patterning.

In the PPR, misexpression of *Otx*2 mRNA results in a loss of *Gbx*2 expression (Fig. 3(E)). To ask whether Otx2 acts as a transcriptional repressor in this context we used a constitutive repressor form *Otx*2*-EnR*. Like full-length *Otx*2, misexpression of *Otx*2*-EnR* leads to *Gbx*2 reduction in the PPR (Fig. 3(F)). Misexpression of *Gbx*2 (Fig. 3(G) and (H)) or the constitutive repressor *Gbx*2*-EnR* (Fig. 3(K) and (L)) reduces *Otx*2 expression in the PPR, while Gbx2 morpholino knock-down (Li et al., 2009) expands its expression (Fig. 3(I) and (J)). Together, these results demonstrate that in the PPR Otx2 and Gbx2 act as transcriptional repressors and mutually repress each other suggesting that this interaction generates the *Gbx*2/*Otx*2 expression boundary.

### Dual Gbx2 function in otic placode specification

**Fig. 4:**
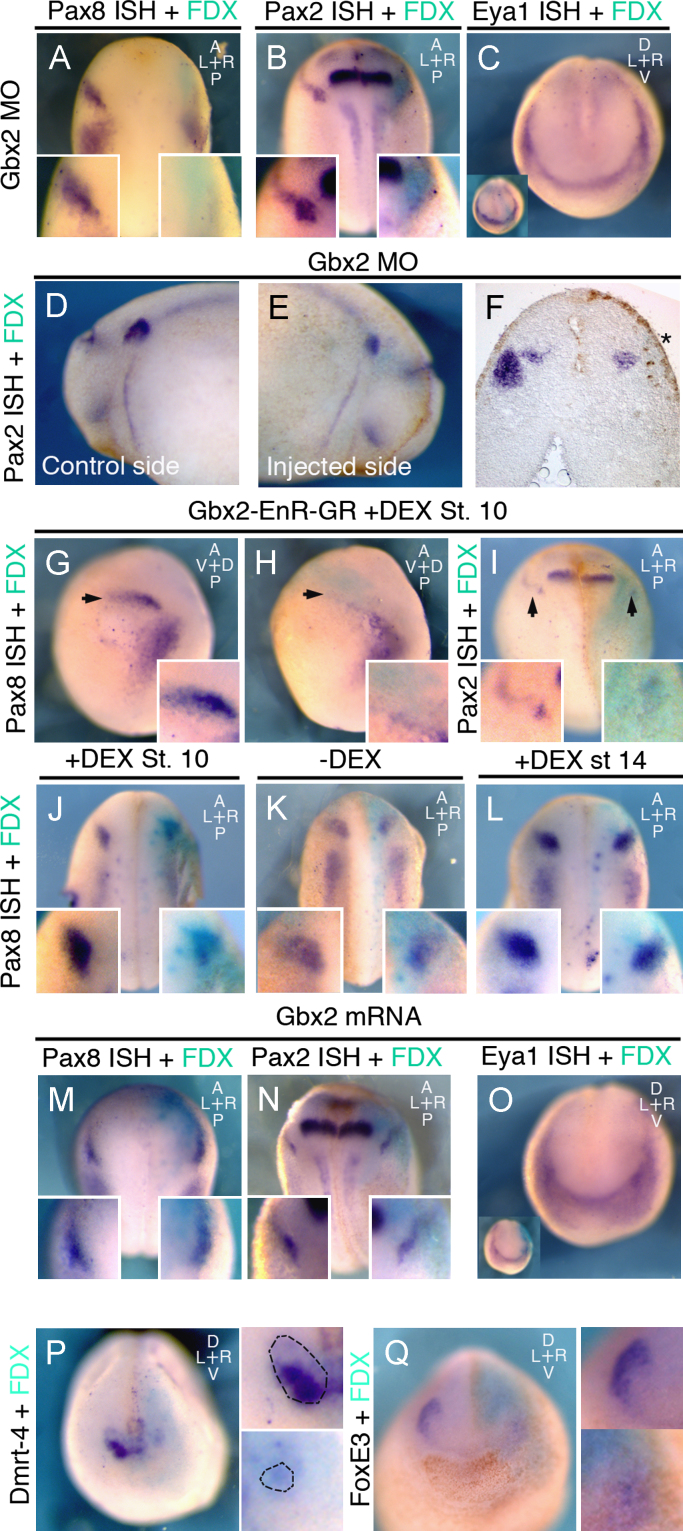
*Gbx*2 is required for otic specification. (A)–(C) Injection of splice and translation blocking Gbx2 morpholinos inhibits otic *Pax*8 (A; 54%, *n*=28) and otic *Pax*2 ((B); 55%, *n*=131; Splice MO: 66% affected, *n*=29; ATG MO: 49% affected, *n*=79). There is no effect on *Eya*1 (0% affected, *n*=30. (C): blue). (D)–(F) At stage 25, *Pax*2 expression is reduced and the otic vesicle is small (asterisk in transverse section F) after injection of splice and translation blocking *Gbx*2 morpholinos ((E) 59% affected, *n*=66; splice MO: 44% affected, *n*=25) when compared to the uninjected side (D). (G)–(I) Injection of inducible *Gbx*2*-EnR-GR*: activation at stage 10 reduces otic *Pax*8 (arrow) at stage 13 ((H); 49%, *n*=43) compared to uninjected side (G) and *Pax*2 at stage 16 ((I); arrow; 46% *n*=18). (J)–(L) Activation of inducible *Gbx*2*-EnR-GR* at stage 10 (J) reduces *Pax*8; no change is observed in absence of DEX ((K); 0% affected, *n*=17) or when DEX is added at stage 14 ((L) 0% affected, *n*=110). (M)–(O) *Gbx*2 mRNA does not expand *Pax*8 ((M); 0% affected, *n*=35), *Pax*2 ((N); 0% affected, *n*=22), and *Eya*1 ((O) 0% affected, *n*=22). (P), (Q) Overexpression of *Gbx*2 reduces *Dmrt-*4 ((P) 66%, *n*=29) and *FoxE*3 ((Q) 92%, *n*=13). Small panels in (P) and (Q) show higher magnification of the control (top) and injected (bottom) side. All embryos were injected into the A3 blastomere at the 32-cell stage; inserts in (A), (B), (G)–(N) high magnification of the otic region. In (C) and (O) turquoise staining reveals FDX. Crosses indicate the orientation of embryos; a: anterior, l: left, p: posterior, r: right, d: dorsal, v: ventral.

The otic placode forms within the *Gbx*2^+^ territory; is Gbx2 required for its specification? Gbx2 knock-down by splice- and translation-blocking morpholinos prevents the expression of the otic marker*s Pax*8 and *Pax*2 (Fig. 4(A) and (B)). When analyzed at later stages, the size of the otic vesicle in embryos injected with both morpholinos is severely reduced (Fig. 4(D) – (F)). Although *Gbx*2 is normally expressed prior to the pre-placodal marker *Eya*1 (Li et al., 2009), *Eya*1 expression is unaffected in Gbx2 morphants (Fig. 4(C)). Thus, Gbx2 is required for otic, but not for PPR specification.

Is this function of Gbx2 simply due to its *Otx*2-repressing activity or does it also regulate otic-specific genes? To test this we used the inducible *Gbx*2*-EnR-GR*, which constitutively represses all Gbx2 targets including *Otx*2 (Fig. 3(K) and (L)). When this construct is activated at the beginning of gastrulation (stage 10), the earliest expression of *Pax*8 (stage 13) and *Pax*2 (stage 16) is reduced (Fig. 4(G)–(I)); *Pax*8 remains absent when embryos are grown to stage 18 (Fig. 4(J)) similar to Gbx2 morphants. In contrast, without activation (Fig. 4(K)) no effect is observed. Thus, even in the absence of Otx2, Gbx2-EnR prevents the expression of otic genes suggesting that the loss of otic markers in Gbx2 morphants is not solely a consequence of ectopic Otx2 expression. To assess whether Gbx2 function is required after initial otic specification, we activated *Gbx*2*-EnR-GR* later at neural plate stages (Fig. 4(L)): otic genes continue to be expressed normally suggesting that Gbx2 function is not required for the maintenance of otic fate.

Finally, we tested whether Gbx2 is sufficient to impart otic character to cells in the anterior PPR. *Gbx*2 misexpression does not lead to expansion or ectopic expression of *Pax*8 (Fig. 4(M)) or *Pax*2 (Fig. 4(N)), nor does it affect the general PPR marker *Eya*1 (Fig. 4(O)). However, ectopic *Gbx*2 expression does repress anterior cell fates as demonstrated by the loss of the olfactory marker *Dmrt*4 (Huang et al., 2005; Fig. 4(P)) and the lens marker *FoxE*3 (Fig. 4(Q)). Thus, while Gbx2 is not sufficient to impart otic identity to non-otic cells, it plays a dual role during otic specification: it restricts *Otx*2 (which otherwise inhibits posterior fate; see below) and provides a positive input for otic specifiers. However, once induced maintenance of otic identity is independent of Gbx2 function.

### Otx2 is required for trigeminal placode specification

**Fig. S1:**
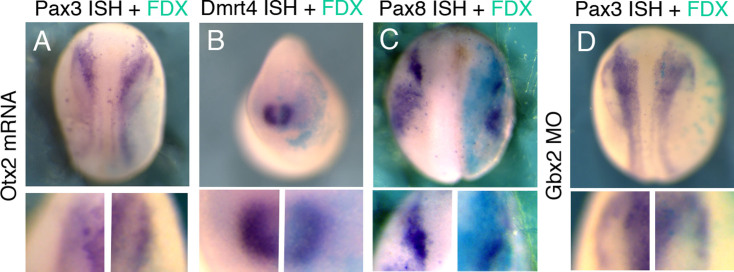


**Fig. 5:**
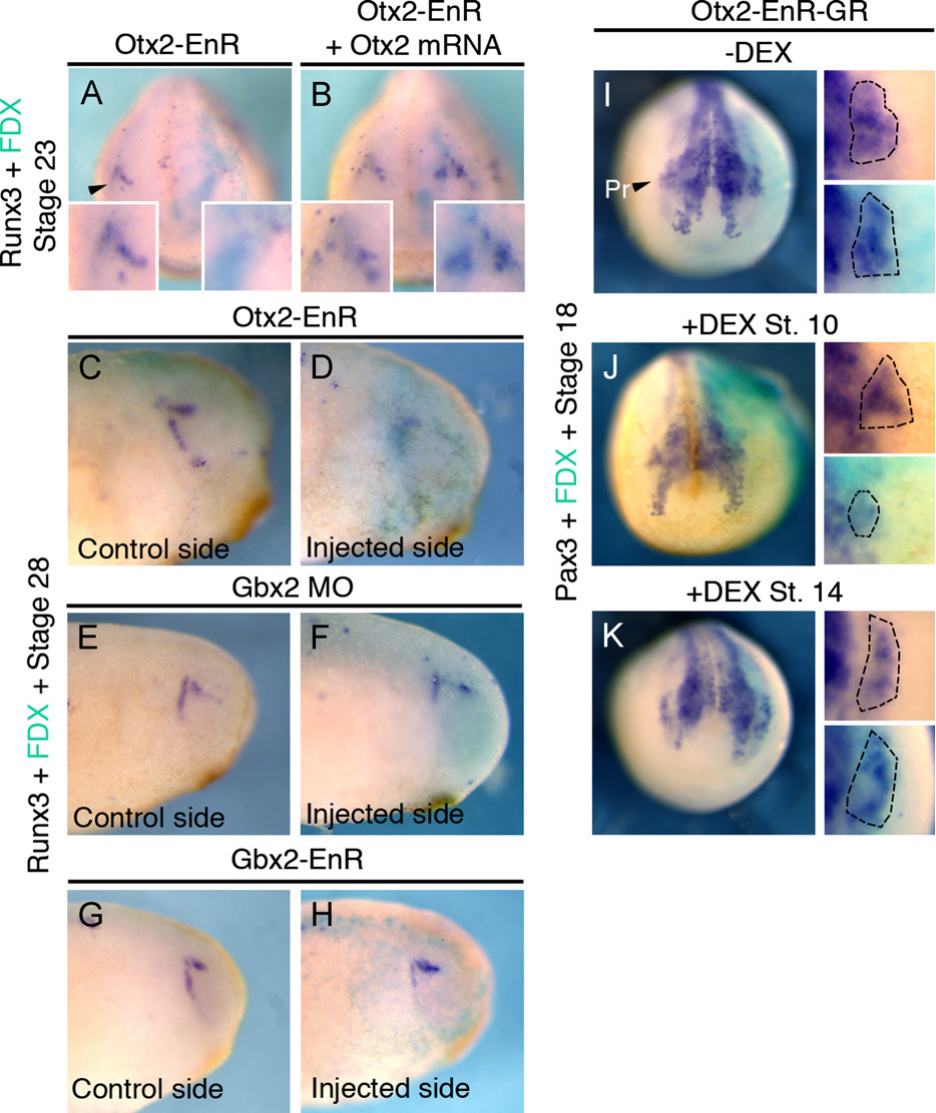
Activation of Otx2 target genes is required for trigeminal placode specification. (A), (B) *Otx*2*-EnR* inhibits *Runx*3 at stage 23 ((A); 59%, *n*=26; arrowhead: trigeminal placode on uninjected side). This is rescued by co-injection of *Otx*2 ((B) inhibition reduced to 17%, *n*=36). Inserts show higher magnification of the trigeminal region. (C), (D) At stage 28 the profundal and trigeminal placodes can be distinguished; both are reduced after *Otx*2*-EnR* injection (78%, *n*=18). Compare control (C) and injected side (D). (E), (F) Injection of *Gbx*2 splice and translation blocking morpholinos ((F) 0% affected, *n*=25) does not affect *Runx*3; compare uninjected (E) and injected side (F). (G), (H). Injection of *Gbx*2*-EnR* does not affect *Runx*3 ((H); 3% affected, *n*=31); compare control (G) and injected side (H). (I)–(K) Activation of *Otx*2*-EnR-GR* at stage 10 inhibits *Pax*3 the profundal placode ((J) 49%, *n*=33); no change is observed without DEX ((I) 5% affected, *n*=61; PR: profundal placode) or when added at stage 14 ((K) 5% affected, *n*=42). Magnifications show profundal region (dotted outline) on the uninjected (top) and injected side (bottom; FDX: turquoise). All embryos were injected into the A3 blastomere at the 32-cell stage.

Future trigeminal cells initially lie within the *Otx*2 domain (Fig. 2(H) and (B)). Is the activation of Otx2 target genes required for trigeminal cell specification? Targeting the PPR with RNA encoding the constitutive repressor form *Otx*2*-EnR* leads to a loss of *Runx*3 labeling trigeminal/profundal precursors (Park and Saint-Jeannet, 2010; Fig. 5(A)) at stage 23, while co-injection with full length *Otx*2 mRNA restores its expression (Fig. 5(B)). To analyze whether activation of Otx2 targets is required for the profundal and trigeminal placode (opV and mmV in amniotes), *Runx*3 expression was assessed at stage 28 when both can be distinguished: after Otx2-EnR injection both placodes are absent (Fig. 5(C) and (D)). Our results suggest that Otx2 and Gbx2 separate prospective otic and trigeminal territories (Fig. 2(B)) predicting that profundal and trigeminal specification should be independent of Gbx2. Indeed, injection of Gbx2 morpholinos (Fig. 5(E) and (F)) or of *Gbx*2*-EnR* (Fig. 5(G) and (H)) does not alter *Runx*3 expression to the same extent as Otx2-EnR, and *Pax*3 expression is normal after Gbx2 knock down (Supplementary Fig. 1D).

To test when the activation of Otx2 target genes is required for trigeminal development, we used inducible *Otx*2*-EnR-GR*. In the absence of DEX, profundal *Pax*3 is normal (Fig. 5(I)); activation at gastrulation stage (stage 10) reduces *Pax*3 (Fig. 5(J)), while activation at stage 14 has no effect (Fig. 5(K)). Otx2 or Otx2E1A (not shown) expression in the trigeminal territory has no effect on *Pax*3 expression (Supplementary Fig. 1A); thus the constitutive repressor form of Otx2 does not mimic misexpression of wild type Otx2 suggesting that Otx2 acts as a transcriptional activator in trigeminal precursors. In addition, ectopic *Otx*2 expression in the posterior PPR is not sufficient to expand *Pax*3 transcripts (Supplementary Fig. 1A). Thus, activation of Otx2 target genes is required early for trigeminal specification, but not late for the maintenance of trigeminal fate.

### An early requirement for *Otx*2 in lens and olfactory placode specification

**Fig. 6:**
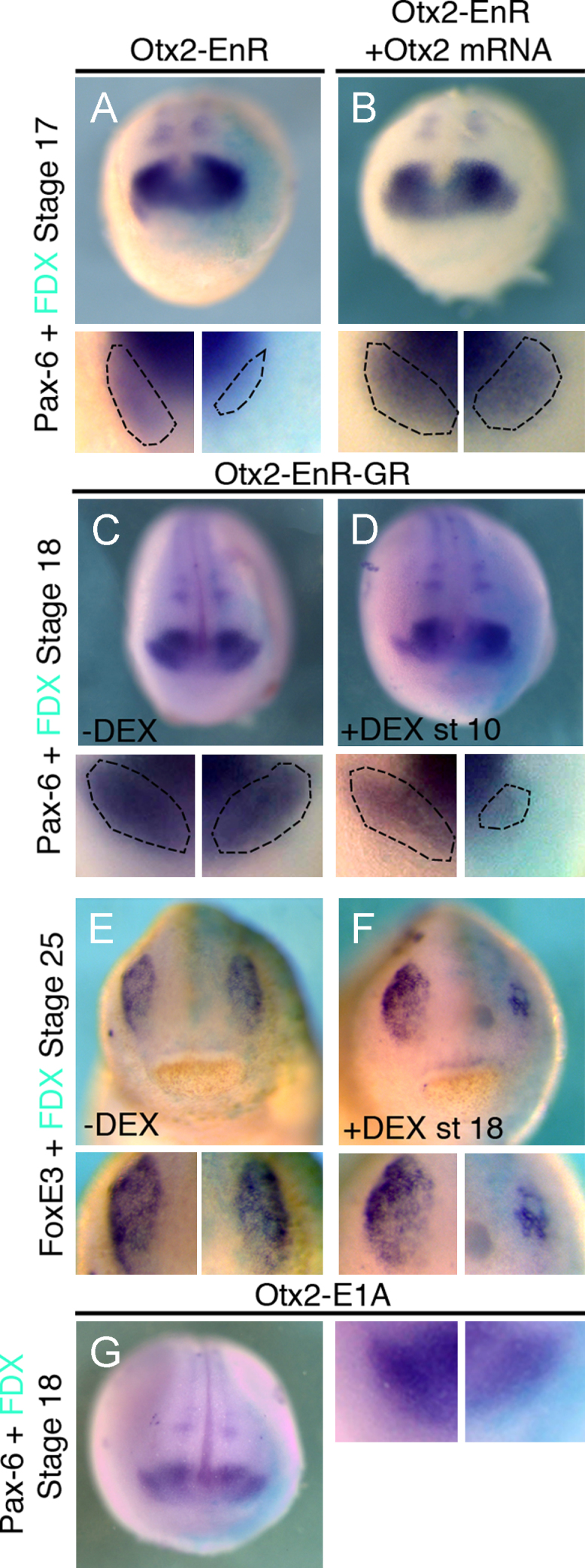
Activation of Otx2 target genes is required at early and late stages of lens placode formation. (A), (B) *Otx*2*-EnR* inhibits *Pax*6 in the lens at stage 17 (50%, *n*=26). This is rescued by co-injection of *Otx*2 ((B); inhibition reduced to 15%, *n*=20). (C)–(F) Activation of *Otx*2*-EnR-GR* at stage 10 reduces lens-specific *Pax*6 at stage 18 ((D) 52%, *n*=25); without DEX *Pax*6 expression is normal ((C) 0% affected, *n*=23). *FoxE*3 at stage 25 is normal without DEX ((E) 0% affected, *n*=30), while addition of DEX at stage 18 leads to reduction ((F) 64%, *n*=22). (G) *Otx*2*-E*1*A* activates Otx2 targets but does not affect lens *Pax*6 (0% affected, *n*=25). All embryos were injected into the A3 blastomere at the 32-cell stage and are shown in frontal view with dorsal to the top. High magnifications of the lens region are shown below each panel; dotted lines demarcate placodal *Pax*6. Turquoise staining reveals the lineage tracer FDX.

**Fig. 7:**
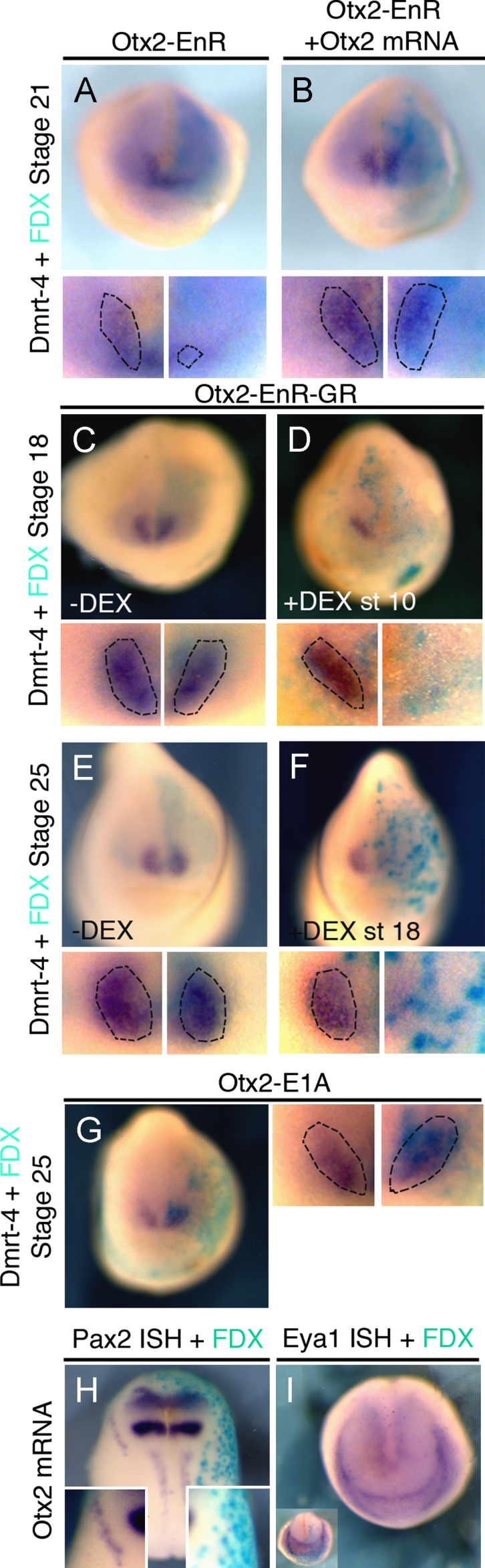
Activation of Otx2 target genes is required at early and late stages of olfactory placode formation. (A), (B) *Otx*2*-EnR* inhibits the olfactory placode marker *Dmrt*4 at stage 21 (59%, *n*=29). This is rescued by co-injection of *Otx*2 ((B) inhibition reduced to 12%, *n*=33). (C–F) *Otx*2*-EnR-GR* injections: in the absence of DEX *Dmrt*4 expression is normal ((C) 5% affected, *n*=20); when DEX is added at stage 10 *Dmrt*4 expression is lost at stage 18 ((D) 59%, *n*=44). At stage 25 *Dmrt*4 is normal in absence of DEX ((E) 8% affected, *n*=26), while activation at stage 18 strongly reduces *Dmrt*4 ((F); 63%, *n*=24). (G) *Otx*2*-E*1*A* has no effect on *Dmrt*4 (0% affected, *n*=14). (H)–(I) *Otx*2 mRNA reduces *Pax*2 ((H) 72%, *n*=25), but does not change *Eya*1 ((I) 0% affected, *n*=44). All embryos were injected into the A3 blastomere at the 32-cell stage and are shown in frontal view with dorsal to the top. High magnifications of the olfactory region are shown in small panels; dotted lines demarcate placodal *Dmrt*4. Turquoise staining reveals the lineage tracer FDX.

*Otx*2 transcripts are present in the anterior PPR including in lens and olfactory precursors and remain expressed once the placodes have formed. While a role for Otx2 has been demonstrated for late lens development (Ogino et al., 2008), its early requirement for either placode has not been investigated. When mRNA encoding the repressor form *Otx*2*-EnR* is injected into the A3 blastomere at the 32-cell stage the early lens marker *Pax*6 (Fig. 6(A)) and the olfactory marker *Dmrt*4 (Fig. 7(A)) are strongly reduced. The loss of both markers is rescued by co-injection with full length *Otx*2 mRNA (*Pax*6: Fig. 6(B); *Dmrt*4: Fig. 7(B)). Activation of inducible *Otx*2*-EnR-GR* at the beginning of gastrulation results in a reduction or loss of both *Pax*6 (Fig. 6(D)) and *Dmrt*4 (Fig. 7(D)), while no effect is observed without DEX (*Pax*6: Fig. 6(C); *Dmrt*4: Fig. 7(C)). Activation of Otx2 target genes is also required later at placode stages: *Otx*2*-EnR-GR* activation at stage 18 results in a complete absence of *Dmrt*4 in the olfactory region (Fig. 7(F)) and down-regulation of *FoxE*3 in the lens (Ogino et al., 2008; Fig. 6(F)) while their expression is normal without DEX (*FoxE*3: Fig. 6(E); *Dmrt*4: Fig. 7(E)). Thus, activation of Otx2 target genes is required for early specification and maintenance of lens and olfactory fates.

Otx2 alone cannot induce ectopic lenses (Ogino et al., 2008); we confirm this using the constitutive activator form of Otx2-E1A (Fig. 6(G)). The same holds true for the olfactory placode: misexpression of Otx2 (Supplementary Fig. 1B) or *Otx*2*-E*1*A* does not expand *Dmrt*4 expression (Fig. 7(G)). However, when misexpressed in the posterior placode territory Otx2 mRNA represses otic fates as indicated by the absence of both *Pax*8 (Supplementary Fig. 1C) and *Pax*2 (Fig. 7(H)) while the general PPR marker *Eya*1 remains unaffected (Fig. 7(I)). Thus, while ectopic *Otx*2 represses posterior placodes and activation of its targets is required for olfactory and lens development, alone it cannot impart anterior fate to posterior the PPR.

## Discussion

### Otx2 and Gbx2 in global ectodermal patterning

To form a functional nervous system its peripheral and central components must develop in register. In the head, the olfactory bulb, the retina and the targets and proximal parts of the sensory ganglia are derived from the central nervous system, while the olfactory epithelium, the lens, inner ear and distal cranial ganglia arise in the non-neural ectoderm from specialized structures, the sensory placodes. How is anterior-posterior patterning between both territories integrated? During development sensory placode precursors originate in the pre-placodal region, where cells of different fates are initially intermingled (Kozlowski et al., 1997; Streit, 2002; Bhattacharyya et al., 2004; Xu et al., 2008; Pieper et al., 2011; for review: Streit, 2007; Schlosser, 2010). Over time, they acquire distinct rostro-caudal identity leading to the alignment with their central counterparts suggesting that a global patterning mechanism imparts positional information to the entire ectoderm (see also: Wada et al., 2006; Patthey et al., 2008). Here we show that the transcription factors Otx2 and Gbx2 are important components of such a mechanism. In the PPR, they segregate otic and trigeminal progenitors (this study), while they establish a compartment boundary at the MHB in the neural plate and prevent mixing of cells with different fates (Millet et al., 1999; Katahira et al., 2000; Tour et al., 2002a; Glavic et al., 2002; Zervas et al., 2004; Sunmonu et al., 2011). In both regions, Otx2 and Gbx2 seem to play a dual role: they repress each other to endow cells with unique identities and to suppress the alternative fate (trigeminal vs otic; midbrain vs rhombomere1), while simultaneously mediating sorting. Initially, both genes partially overlap and mutual repression at the transcriptional level is likely to form a gene expression the boundary. Subsequently, cell sorting ensures compartmentalization to restrict cells of the same fate to a contiguous domain. Accordingly, in the brain, Otx2 deficient cells segregate from wild type neighbors as do cells expressing exogenous Otx2 in rhombomere (Rhinn et al., 1998; Sunmonu et al., 2011). Likewise, our results show that *Otx*2 and *Gbx*2 expressing cells sort out in the non-neural ectoderm. The degree of cell mixing in the placode territory is still under debate with more cell mixing observed in chick than in *Xenopus* (Streit, 2002; Bhattacharyya et al., 2004; Xu et al., 2008; Pieper et al., 2011). As fate maps may introduce some error due to variability between different embryos, ultimately live imaging over long time periods will be required to resolve this question. Nevertheless, together with previous studies on neural and neural crest cells (Wassarman et al., 1997; Acampora et al., 1995, 1997, 1998; Rhinn et al., 1998; Broccoli, et al., 1999; Li et al., 2005; Li et al., 2009; Sunmonu et al., 2011) our findings establish cross-regulatory interactions between Otx2 and Gbx2 as key components for global ectodermal patterning. Both factors establish anterior-posterior identity across the embryonic ectoderm and mediate cell sorting to segregate cells of different fates.

These observations also suggest that signals that establish anterior-posterior identity (for review: Wilson and Houart, 2004) not only pattern the neural plate, but the entire ectoderm with transcription factors like Otx2 and Gbx2 as a read-out. Among these Fgfs, Wnts, Retinoic Acid, Nodals and BMPs provide posteriorizing factors, while their antagonists protect anterior identity. Indeed, elevated Wnt activity in zebrafish leads to an expansion of posterior neural and placodal fates (Kim et al., 2000; Heisenberg et al., 2001). Wnt signaling also promotes derivatives of the posterior neural plate border, neural crest cells, and Gbx2, a direct Wnt target, mediates its activity (Chang, Hemmati-Brivanlou, 1998; Bang et al., 1999; Villanueva et al., 2002; García-Castro et al., 2002; Bastidas et al., 2004; Basch et al., 2006; Patthey et al., 2008; Steventon et al., 2009; Li et al., 2009). In addition to such global patterning mechanisms local signaling and downstream transcriptional networks subsequently fine tune allocation of different cell fates.

### Patterning the placode territory

In the PPR, the *Otx*2/*Gbx*2 boundary roughly separates prospective otic and trigeminal fates suggesting that olfactory, lens and trigeminal precursors receive different transcriptional inputs from otic progenitors. The transcriptional regulation of the PPR marker *Six*1 supports this idea. Although *Six*1 is expressed in a contiguous domain containing all sensory progenitors, different enhancers control its expression along the anterior-posterior axis (Sato et al., 2010). Cells from the anterior *Six*1 domain contribute to the olfactory, lens and trigeminal placodes, but not to the otic. These findings suggest that one of the first subdivisions of the placode territory occurs between trigeminal and otic precursors clearly grouping trigeminal precursors together with other anterior placodes unlike an earlier suggestion to group profundal and trigeminal placodes in *Xenopus* with posterior progenitors (Schlosser, 2006). Shortly thereafter, the PPR begins to express other transcription factors in nested domains to subdivide this territory further (for review: Schlosser, 2006).

*Otx*2 and *Gbx*2 are already expressed at gastrula stages (Simeone et al., 1992, 1993; von Bubnoff et al., 1996; Tour et al., 2001) and act early during placode specification. Gbx2 is required for the onset of otic-specific genes, where it appears to act as transcriptional activator: the constitutive repressor Gbx2-EnR mimics MO-mediated knock-down. This is in contrast to its earlier role as repressor during boundary formation (see above) suggesting that the availability of cofactors determines the final outcome as observed for other homeobox factors (Brugmann et al., 2004; Anderson et al., 2012). After initial specification, otic development is Gbx2 independent, although it is later involved in ear morphogenesis (Lin et al., 2005). The lack of an early ear phenotype in Gbx2 mutant mice (Lin et al., 2005) is likely due to functional redundancy with Gbx1. In contrast, Otx2 is necessary for both formation and maintenance of lens and olfactory identity (Ogino et al., 2008; this work) consistent with its continued expression in both placodes. In the trigeminal placode, *Otx*2 is downregulated shortly after its specification probably due to repression by Pax3 (unpublished observations), which also inhibits *Pax*6 in this territory (Wakamatsu, 2011). Like Gbx2, Otx2 switches from a transcriptional repressor at early stages to an activator later. In summary, like in the neural plate, in the PPR Otx2 and Gbx2 are among the earliest factors that subdivide a contiguous territory along the anterior-posterior axis.

Although Otx2 and Gbx2 are required for early placode specification, neither factor alone is sufficient to endow cells with new regional character or to induce ectopic placodes. This appears to differ considerably from their activity in the neural tube, where ectopic expression of either factor respecifies anterior-posterior identity (Glavic et al., 2002; Tour et al., 2002a). However, here Otx2 and Gbx2 mainly function to position the MHB (Li and Joyner, 2001; Raible and Brand, 2004), an organizer region that itself patterns the brain. Thus, changes in regional identity are likely to be a consequence of MHB induction. Whether a similar organizing center forms at the Otx2/Gbx2 boundary in the PPR remains to be established, however, so far our results argue against this notion. The finding that neither Gbx2 nor Otx2 is sufficient to induce ectopic placodes suggests that additional factors cooperate to control the expression of placode-specific downstream targets. This is indeed the case in the lens, where *Otx*2 directly binds to the lens-specific *FoxE*3 enhancer and together with intracellular effectors of Notch signaling activates its transcription (Ogino et al., 2008).

### Evolutionary conservation of anterior-posterior patterning by Otx2 and Gbx2

The development of cranial sensory placodes and neural crest is considered to be a key step in the evolution of the vertebrate head (Northcutt and Gans, 1983). Like in vertebrates *Gbx* and *Otx* form a boundary within the *Amphioxus* ectoderm (Williams and Holland, 1996, 1998; Castro et al., 2006; Benito-Gutiérrez, 2006) raising the question whether, at an early stage of their evolution, neural crest and placodes co-opted an already existing gene expression boundary to position themselves along the anterior-posterior axis. Despite *Gbx*2/*Otx*2 apposition in *Amphioxus*, MHB specific genes such as *En*, *Wnt*1, *FGF*8/17/18 and *Pax*2/5/8 are not restricted to this boundary (Holland et al., 1997, 2000; Meulemans and Bronner-Fraser, 2007), indicating that MHB organizer genes were recruited to the Otx/Gbx border in early vertebrates (Castro et al., 2006; Holland and Short, 2008; Holland, 2009). A Gbx/Otx boundary appears to have been present in the early bilaterian ancestor as *Unpg/Gbx* and *Otd/Otx* also negatively regulate one another to form a boundary that positions *En* and *Pax*2*/*5*/*8 in *Drosophila* (Hirth et al., 2003). In addition, *Gbx*2 and *Otx*2 form a boundary in the annelid *Platynereis dumerilii* that corresponds to a band of En expression (Arendt et al., 2001; Steinmetz et al., 2007, 2011). Together these findings raise the possibility that Otx2 and Gbx2 form an ancient boundary of gene expression responsible for anterior-posterior patterning of both the neural plate and neural plate border. However, this boundary has been utilized differently in each territory: to position an organizing region at the MHB, and to specify placodal fates in the PPR.
